# Are Mitochondria a Potential Target for Treating β-Thalassemia?

**DOI:** 10.3390/jcm14041095

**Published:** 2025-02-08

**Authors:** Elena Di Pierro, Valeria Di Stefano, Margherita Migone De Amicis, Giovanna Graziadei

**Affiliations:** SC di Medicina ad Indirizzo Metabolico, Fondazione IRCCS Ca’ Granda Ospedale Maggiore Policlinico, Via Francesco Sforza 28, 20122 Milano, Italy; valeria.distefano@policlinico.mi.it (V.D.S.); margherita.migone@policlinico.mi.it (M.M.D.A.); giovanna.graziadei@policlinico.mi.it (G.G.)

**Keywords:** β-thalassemia, mitochondria, ineffective erythropoiesis

## Abstract

The inherited genetic disorder β-thalassemia affects the hematopoietic system and is caused by the low production or absence of adult hemoglobin (HbA). Ineffective erythropoiesis is the hallmark of β-thalassemia pathophysiology and is characterized by an erythropoietin-driven substantial increase in erythroblast proliferation, coupled with an increase in late-stage precursor apoptosis, which results in low levels of circulating mature red blood cells (RBCs) and chronic anemia. Mitochondrial dysfunction commonly occurs in these cells because of the increased demand for energy production and the need to manage abnormal hemoglobin chain synthesis. Moreover, several studies have highlighted the importance of gradual mitochondrial clearance for mature erythroid cell production. This review offers an overview of the mitochondrial role in essential cellular processes, particularly those crucial for maintaining RBC health and function. Additionally, recent evidence regarding the contribution of mitochondrial dysfunction to the pathophysiology and severity of β-thalassemia is discussed, along with updated insights into indirect mitochondria-targeting treatments, which present potential pharmacological targets.

## 1. Introduction

Thalassemia is the most prevalent genetic autosomal recessive disorder globally, affecting about 1.5% of the population at carrier level. It is characterized by the abnormal synthesis of hemoglobin, leading to anemia [1,2]. The evolutionary relationship between the thalassemia carrier state and malaria resistance accounts for its high frequency in regions spanning from sub-Saharan Africa, the Middle East, and the Mediterranean to Southeast Asia. However, the prevalence of the disease has been rising in other regions, including Northern Europe and North America, primarily due to migration [3].

Specifically, β-thalassemia is caused by the absence of or a reduction in the synthesis of β-globin chains. The imbalance of α/β globin chains and the relative excess of unpaired α-globin chains results in the formation of cytotoxic intracellular precipitates that compromise erythroid cell production and maturation in the bone marrow, leading to ineffective erythropoiesis, the hallmark of β-thalassemia pathophysiology [4]. Moreover, oxidative stress and membrane damage in circulating erythroid cells lead to chronic hemolysis, which leads to the release of free hemoglobin (Hb) and free heme in the bloodstream [5]. Both ineffective erythropoiesis and increased hemolysis lead to deregulated iron homeostasis in patients, with the release of erythroid factors that suppress hepcidin production and cause iron overload, which is also worsened by regular transfusions [6].

The clinical manifestations of the disease are extremely heterogeneous, varying from very mild to more severe forms, called non-transfusion-dependent thalassemia (NTDT) and transfusion-dependent thalassemia (TDT), respectively [7]. The considerable variability and severity in clinical expression depend on the specific genotypes due to different β^+^ or β° alleles, known as primary modifiers, and on the co-presence of other independent genetic factors that may worsen or alleviate the phenotype [8]. However, individuals with identical β-thalassemia genotypes can have variable clinical severities [9], because of the high complexity of the genetic background associated with the disease [10] or because of acquired and ambient factors [11].

Two secondary key modifiers, i.e., the coinheritance of α-thalassemia and the innate ability to produce fetal hemoglobin (HbF), can ameliorate the imbalance of α/β globin chains, affecting the pathophysiology of β-thalassemia at the primary level [12]. Common DNA polymorphisms and rare mutations in cis-regulatory elements modulating expression of the *HBG2* [13,14], *BCL11A* [15], *HBS1L* [16], *MYB* [17,18], and *KLF1* [19] genes may be associated with a delay in the fetal-to-adult hemoglobin switch and/or an increase in the synthesis of HbF in adults [20]. Other studies focusing on different human populations reported that these single nucleotide polymorphisms (SNPs) are deeply geographically structured and that other loci, including *KCNK10*, *GPR65*, *RNASE2*, *RNASE3*, and *C/EBPE*, are also involved in the regulation of HbF expression [21,22].

**Figure 1:**
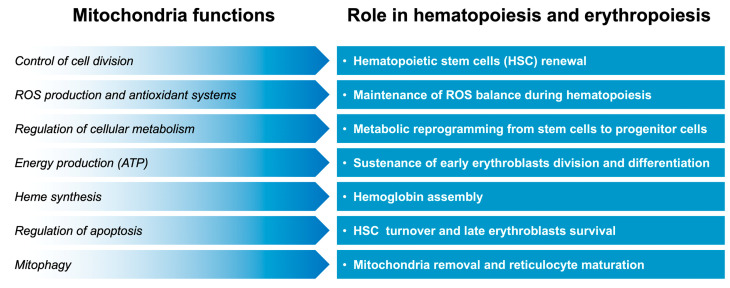
Overview of mitochondria functions in maintaining red blood cell production and function.

The upregulation of peroxisome proliferator-activated receptor γ coactivator-1α (PGC-1α) can induce fetal hemoglobin synthesis in human primary erythroblasts [23]. PGC-1α belongs to a family of coactivator proteins that play a key role in regulating various signaling pathways [24]. Along with its effects on the liver, neurons, and muscle [25], studies have suggested that PGC-1α also plays an important role in the maturation and survival of erythroid cells [26]. PGC-1α also regulates mitochondria biogenesis and functions [27]. An increase in mitochondrial activity facilitated by increased PGC-1α levels can create a cellular environment conducive to erythropoiesis and the synthesis of hemoglobin, including HbF. In another study, PGC1β-depleted erythroid progenitor cells showed an increase in mitochondrial mass, suggesting impaired mitochondrial clearance [28]. These findings suggest that mitochondria play a crucial role in β-thalassemia pathophysiology and also contribute to β-thalassemia-related complications. In this review, we summarized evidence concerning the role of mitochondria, especially in maintaining red blood cell production and function (Figure 1).

## 2. Mitochondria in Physiology

Mitochondria are essential organelles in eukaryotic cells because they play an important role in producing adenosine triphosphate (ATP) through oxidative phosphorylation (OXPHOS) and providing energy for the activities in the cell [29]. They also play critical roles in several other cellular functions, including the regulation of different metabolic pathways (such as glycolysis, beta-oxidation, and amino acid metabolism) to produce ATP, which is crucial for nearly all cellular activities [30,31]. Moreover, mitochondria control intracellular calcium levels, which are crucial for cellular signaling, muscle contraction, neurotransmitter release, and cell division [32]. They also modulate cell survival by regulating programmed cell death. When a cell is damaged or stressed, mitochondria can release proteins such as cytochrome c into the cytoplasm, which activates caspases—enzymes that drive apoptosis. This ensures that damaged or unnecessary cells are efficiently eliminated, maintaining tissue homeostasis [33].

In certain cells, such as those in brown adipose tissue, mitochondria can produce heat instead of ATP through thermogenesis [34]. This process involves the uncoupling of oxidative phosphorylation, where energy created by the electron transport chain is released as heat instead of being used to produce ATP. This process helps regulate body temperature, especially in newborns and during cold exposure [35]. Mitochondria also support biosynthetic pathways, such as those associated with the synthesis of steroid hormones, including glucocorticoids, sex hormones, and mineralocorticoids, primarily in endocrine cells. The enzymes required for these processes are located in the mitochondrial membrane and convert cholesterol into steroid precursors [36].

Mitochondria can replicate and increase in number in response to the energy demands of the cell through a process called mitochondrial biogenesis [37,38]. This involves the activation of transcription factors such as PGC-1α, which stimulate the expression of genes related to mitochondrial function [24]. This process is important in cells with high-energy demands, such as muscle cells and neurons, and helps the metabolism of the cell to adapt to different environmental or energy states [27]. Mitochondria are highly dynamic organelles that can undergo fusion (the combination of two mitochondria) and fission (the splitting of one mitochondrion into two). These processes help maintain mitochondrial health by allowing the exchange of mitochondrial contents and the removal of damaged parts [39]. Fusion helps in the mixing of mitochondrial contents, such as mitochondrial DNA and proteins, which is beneficial for repairing damaged mitochondria. Fission facilitates mitochondrial division and replication, ensuring that cells can produce enough mitochondria to meet their energy needs [40,41].

Moreover, mitochondria can be selectively degraded through mitophagy, a form of autophagy in which damaged or dysfunctional mitochondria are engulfed and broken down by lysosomes in cells. This helps control mitochondrial quality and protect the cell from stress caused by defective mitochondria [42]. Mitochondria are the primary site of reactive oxygen species (ROS) production as byproducts of the electron transport chain. Although ROS can damage cellular components such as proteins, lipids, and DNA, they also play roles in cell signaling and defense against pathogens [43,44]. Mitochondria have antioxidant systems (such as superoxide dismutase and glutathione peroxidase) to neutralize excess ROS and protect the cell from oxidative damage [45].

Mitochondria participate in the synthesis of iron–sulfur clusters, which are essential for many enzymes involved in cellular metabolism [46]. They also play a central role in heme synthesis, which is crucial for the production of hemoglobin in red blood cells [47]. Mitochondria help incorporate iron into porphyrin rings to produce heme, a key component of hemoglobin.

## 3. Mitochondria in Pathology

As mitochondria are necessary for cellular activities, their dysfunction leads to various human pathological conditions [48]. Mitochondria have a 16-kilobase mitochondrial genome (mtDNA), which is distinct from the nuclear DNA (nDNA) found in the nucleus of cells. This DNA is circular and encodes only a few proteins that are required for mitochondrial function, most of which are involved in oxidative phosphorylation, whose expression and function are strictly coordinated by the nuclear DNA [49].

Mutations in mtDNA or nDNA that affect mitochondrial function can lead to various inherited diseases, such as Leber’s hereditary optic neuropathy (LHON), Kearns–Sayre syndrome (KSS), mitochondrial encephalomyopathy, lactic acidosis, and stroke-like episodes (MELAS), mitochondrial myopathy, and Leigh syndrome [50,51]. These diseases are often characterized by defects in energy production, leading to tissue dysfunction, particularly in high-energy-demanding organs such as muscles and the brain. Besides lactic acidosis, impaired ATP production results in neurological and muscle problems. The symptoms include muscle weakness, exercise intolerance, fatigue, progressive loss of vision due to degeneration of retinal cells, reduced cardiac contractility, and heart failure [52,53,54].

Mitochondrial dysfunction is also associated with several neurodegenerative diseases, particularly those that involve the central nervous system (CNS), due to the high-energy demands of neurons. These diseases include Parkinson’s disease [55], Alzheimer’s disease [56], Huntington’s disease [57], and amyotrophic lateral sclerosis (ALS) [58]. These conditions often involve mitochondrial damage due to the loss of mitochondrial maintenance systems or the accumulation of proteins that interfere with mitochondrial function, leading to energy deficits and an increase in oxidative stress.

However, mitochondrial functions decline with age, leading to a decrease in ATP production and an increase in ROS production [59]. Moreover, mtDNA is highly vulnerable to oxidative damage due to its proximity to ROS produced in mitochondria [60]. The common 4977-bp mitochondrial deletion (ΔmtDNA4977) increases in frequency with oxidative stress [61] and accumulates in aging tissues, serving as a biomarker of mtDNA damage [62]. This contributes to cellular senescence, tissue degeneration, and age-related diseases, including osteoporosis, macular degeneration, and sarcopenia (muscle loss). The expression of antioxidants can ameliorate age-dependent anemia and decrease ineffective erythropoiesis. This effect is mediated by the activation of the nuclear factor erythroid-2-related factor (Nrf2) function by peroxiredoxin-2, which ensures erythroid maturation and growth during aging [63].

Mitochondria also regulate glucose and lipid metabolism, and their dysfunction can exacerbate metabolic disorders [64]. Additionally, mitochondrial dysfunction is often observed in cancer cells [65]. Excessive ROS production contributes to DNA damage and genomic instability, which is a hallmark of cancer development. Cancer cells may also undergo metabolic reprogramming (known as the Warburg effect) to favor glycolysis over oxidative phosphorylation, even in the presence of oxygen, a process known as aerobic glycolysis. This helps cancer cells produce energy and biosynthetic precursors more rapidly, supporting rapid cell growth [66]. Dysfunctional mitochondria can release damage-associated molecular patterns (DAMPs), which trigger the immune system and contribute to autoimmune diseases such as rheumatoid arthritis and lupus. Finally, mitochondrial stress plays a role in activating inflammatory pathways associated with chronic inflammatory diseases [67].

## 4. Mitochondria in Hematopoiesis and Erythropoiesis

Hematopoiesis involves the differentiation of hematopoietic stem cells (HSCs) into various blood cell lineages, including red blood cells (RBCs), white blood cells (WBCs), and platelets. Mitochondria play crucial roles in this process, considering that they are involved in energy production, regulation of cellular metabolism, and maintenance of cell survival [68]. During hematopoiesis, the shift from stem cells to differentiated progenitor cells and specialized blood cells is accompanied by metabolic reprogramming [69], which is orchestrated by mitochondria to meet the energy and biosynthetic demands of each type of cell [70,71]. Early-stage hematopoietic progenitor cells rely more on glycolysis (anaerobic metabolism) for energy, but as they mature into specialized blood cells, they increasingly rely on oxidative phosphorylation, which is a more energy-efficient process [72]. The early commitment of the erythroid lineage can be affected via the block of mitochondrial respiration [73,74].

In hematopoietic cells, mitochondrial dynamics are also important for cell division and differentiation. Fusion facilitates the mixing of mitochondrial contents, helping cells cope with metabolic stress, whereas fission enables the production of new mitochondria to support rapidly dividing cells [75]. Moreover, the regulation of apoptosis, a type of programmed cell death, is especially important in hematopoiesis, where proper cell turnover is necessary. This process ensures that only healthy cells survive, maintaining the proper balance of blood cell populations. Mitochondrial dysfunction can result in defects in the self-renewal and differentiation of HSCs, potentially leading to bone marrow failure or hematological diseases such as leukemia [76]. Additionally, the ROS generated in mitochondria regulate the self-renewal and differentiation of HSCs. Low ROS levels can stimulate the function and differentiation of HSCs, whereas excessive ROS can cause DNA damage and lead to hematopoietic cell death. Thus, mitochondria help maintain a balance of ROS that supports normal hematopoiesis [77].

Erythropoiesis is the process by which RBCs are produced. This process occurs mainly in the bone marrow and involves several stages of proliferation, differentiation, and terminal maturation from erythroid progenitor cells to highly specialized oxygen-transporting cells filled with hemoglobin (erythrocytes). Mitochondria play a crucial role, particularly in the early phases of erythropoiesis. Early erythroblasts (precursors to red blood cells) rely on mitochondria for energy as they undergo rapid division and differentiation [70]. At this stage, mitochondrial biogenesis, respiration, and metabolism are enhanced at least partly through mTORC1 (mammalian target of rapamycin complex 1)-mediated protein translation [78]. A mitochondrial deficiency induced by the loss of a major mitochondrial transcriptional regulator, mitochondrial transcription factor A (TFAM), in erythroid cells leads to the inhibition of class I and II histone deacetylases (HDACs), causing increased histone acetylation and persistent expression of HSPC-associated genes to impair erythroid differentiation. These findings are consistent with the role of mitochondria in the modulation of key metabolites associated with epigenetic regulation [74]. Similarly, defective mitochondrial function impairs erythroid cell differentiation and leads to anemia due to the insufficient production of mature red blood cells [79]. Moreover, conditions such as mitochondrial myopathy or Leber’s hereditary optic neuropathy are associated with defects in mitochondrial DNA that affect blood cell production [80].

As erythroblasts mature, their mitochondria become less active, and the cells start extruding their nuclei (reticulocytes) and finalizing their maturation into erythrocytes, which lack mitochondria. The molecular pathways involved in mitochondrial degradation in reticulocytes need to be fully elucidated, although researchers have made several important findings regarding this process, indicating that autophagy is important for mitochondrial clearance during terminal erythroid maturation [81]. NIX and ULK1, two critical regulators of autophagy, play major roles in organelle clearance during reticulocyte maturation [82,83]. Nutrient and energy deprivation can result in the activation of ULK1 and autophagy induction by two sensor molecules, mTOR and AMP-activated protein kinase (AMPK) [84]. An increase in autophagy leads to a decrease in apoptosis during β-thalassemic mouse and patient erythropoiesis, suggesting that a lack of autophagy might be associated with the high apoptosis of erythroblasts, a hallmark of ineffective erythropoiesis [85]. The loss of autophagy in erythroid cells leads to the defective removal of mitochondria and severe anemia in vivo. This is important since abnormal RBC retention in damaged mitochondria is responsible for the high frequency of death of erythrocytes [86]. Moreover, analyses of transcriptomic, proteomic, and metabolomic changes between hematopoietic stem/progenitor cells and differentiating erythroid cells in mice and humans have shown that mitochondria are critical regulators of erythropoiesis and participate in erythroid cell maturation, erythroblast enucleation, and RBC production [87].

Finally, the key function of mitochondria during erythropoiesis involves the synthesis of heme, the iron-containing molecule that binds oxygen in RBCs. Mitochondria are closely involved in the production of porphyrins, which are then used to produce heme. However, mitochondria also participate in iron metabolism, which must be regulated to prevent excess iron accumulation, as it can be toxic to cells. Mitochondrial iron is required for heme synthesis, and the proper handling of iron is essential for the production of hemoglobin and the development of functional red blood cells [79].

## 5. Mitochondria in Sickle Cell Disease

Sickle Cell Disease (SCD) refers to a group of inherited hemoglobinopathies that result in the production of hemoglobin S (HbS), a specific structural variant of the β-globin chain. SCD encompasses conditions caused by homozygosity for the HbS variant or compound heterozygosity with other hemoglobin variants such as HbC or with a β-thalassemia allele. This abnormality leads to the transformation of red blood cells (RBCs) into a rigid, sticky, crescent-shaped form, impairing their capacity to transport oxygen effectively and causing various health issues [88].

Recent studies have increasingly identified mitochondria as central components in the pathophysiology of SCD [89]. Interestingly, mature RBCs from individuals with SCD have been found to retain functional mitochondria, a deviation from the normal maturation process where mitochondria are usually discarded [90]. This feature has been observed in both human patients and animal models, linked to compromised mitophagy or stress erythropoiesis [91]. The retention of mitochondria in RBCs is directly associated with the number of reticulocytes, thus linking mitochondrial retention with hemolysis in SCD patients and an increased oxygen consumption rate in SCD RBCs [92]. This retention contributes to higher oxidative stress within the cells, as active mitochondria produce reactive oxygen species (ROS). Elevated ROS levels can cause damage to cellular structures, accelerating premature RBC senescence and hemolysis, thereby worsening anemia and other SCD-related complications [93]. Moreover, the presence of functional mitochondria in mature sickle RBCs has been associated with a greater likelihood of these cells undergoing sickling in low-oxygen environments [94]. This sickling reduces RBC flexibility and increases cell fragility, contributing to the vaso-occlusive events characteristic of SCD.

In addition, SCD patients have been found to have elevated levels of cell-free mitochondrial DNA (cf-mtDNA) in their bloodstream. This cf-mtDNA functions as a damage-associated molecular pattern (DAMP), triggering inflammatory responses and promoting the formation of neutrophil extracellular traps (NETs) [95]. These inflammatory responses further enhance the chronic inflammation seen in SCD patients. In conclusion, the abnormal retention of functional mitochondria in mature RBCs and the increased levels of cf-mtDNA in circulation are key contributors to oxidative stress, inflammation, and the complex pathophysiology of sickle cell disease.

## 6. Mitochondria in β-Thalassemia

In contrast, the relationship between mitochondria and β-thalassemia remains largely unclear. The excess globin chains that accumulate in red blood cell precursors can directly interact with mitochondria, impairing their function. Moreover, the inefficient production of hemoglobin causes an erythropoietin-driven expansion of early-stage erythroid precursors, leading to an increase in metabolic load [70]. This can cause mitochondrial dysfunction, as these cells support the increased energy demands related to the ineffective production of red blood cells [96].

When mitochondria become dysfunctional, they produce excessive ROS, leading to oxidative stress. ROS can further damage mitochondrial membranes, proteins, and DNA, impairing general cell function. A substantially different redox state, resulting from the differing levels of EPO, was reported in newly isolated CD34+ cells from β-thalassemia/Hb E patients compared to those from normal controls [97]. The effects on mitochondria were observed by the seventh day of differentiation, and significant deficits in activity were observed on day 10, coincident with significant levels of globin chain synthesis [98]. The greater number of mitochondria present in cells from thalassemia patients on day 10 suggested that the effect was magnified, with the damage to mitochondria at this point being coincident with the onset of apoptosis and ineffective erythropoiesis [99]. A significantly higher autophagic activity was observed in β-thalassemia/HbE erythroblasts compared to normal control erythroblasts during erythropoiesis [100]. Autophagy has been shown to alleviate the accumulation of excess α-globin precipitates in erythroblasts. This increase is considered a cellular adaptation aimed at reducing the toxicity of excess unbound α-globin chains by degrading these proteins and preventing apoptosis. Furthermore, mitophagy, the selective autophagic degradation of mitochondria, has been found to be enhanced during erythroid differentiation in β-thalassemia [101]. The elevated expression of genes involved in erythroid mitophagy, such as *BNIP3L* and *PINK1*, was also noted in β-thalassemia/HbE erythroblasts [102]. This increase in mitophagy aids in the clearance of damaged mitochondria, which is essential for proper RBC maturation. However, if the protective autophagy process becomes overwhelmed by the persistent stress caused by unpaired α-globin chains, excessive autophagy in β-thalassemia erythroblasts may contribute to elevated apoptosis, impairing erythropoiesis. Indeed, delayed erythroid maturation and signs of impaired mitochondria clearance were observed in β-thalassemia/HbE cells at the terminal stages of differentiation [102].

**Figure 2:**
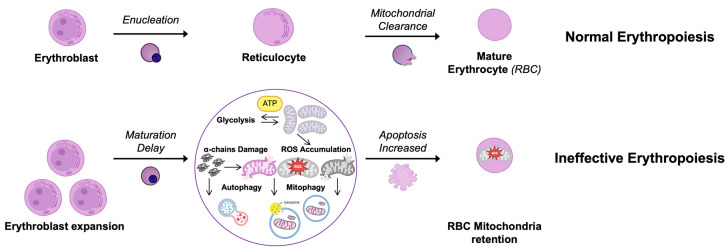
Role of mitochondria in Thalassemia. The upper panel shows normal erythropoiesis, the bottom panel the ineffective erythropoiesis. In β-thalassemia, the expansion of erythroblasts leads to an increase in energy demand requiring mitochondria for ATP production. This causes a delay in enucleation and mitochondrial dysfunctions with consequent ROS accumulation and mitochondrial damage. Moreover, the excess of α-globin chains can directly damage mitochondria, impairing their function. Both autophagy and mitophagy are stimulated in order to reduce the toxicity of α-globin chains and damaged mitochondria, respectively. However, an increase in apoptosis and in mature RBC mitochondrial retention is observed.

These findings highlight the importance of erythroid maturation and suggest that restoring terminal maturation could be a potential therapeutic target for β-thalassemia. However, additional research is needed to confirm this concept and explore its potential for developing new treatment approaches (Figure 2).

Moreover, a marked increase in mtDNA to nuclear DNA copy number (Mt/N) and ΔmtDNA4977 was observed in blood cells from adult individuals with transfusion-dependent thalassemia [103]. These findings supported an adaptive mechanism to secondary oxidant stress from uncontrolled labile plasma iron [104]. However, as in sickle cell disease (SCD), the retention of functional mitochondria in mature erythroid cells becomes a source of ROS, which further causes mtDNA damage and cell degradation. Similarly, stress erythropoiesis or defects in mitophagy may also be the source of mitochondrial retention in β-thalassemia.

## 7. Mitochondria-Targeting Therapy

Although mitochondrial-targeted therapies are not currently being tested in clinical trials for thalassemia, ongoing research continues to examine various aspects of the disease, including mitochondrial dysfunction, with the aim of improving patient outcomes. The recently approved drug luspatercept (ACE-536), which acts as a ligand trap for TGF β-like molecules, increases the differentiation of late erythroblasts, reduces hemichromes, and ameliorates anemia in a dose-dependent manner [105,106]. However, the metabolic pathways relevant to disease pathophysiology and the underlying mechanisms are poorly understood. In contrast, Mitapivat, a pyruvate kinase activator, has positive metabolic effects on animal models and patients [107]. The oral administration of drugs was found to ameliorate ineffective erythropoiesis in β-thalassemia model mice, resulting in increased ATP levels, reduced ROS levels, and improved mitochondrial clearance [108,109].

Moreover, early studies using a mouse model of SCD showed that the inhibition of lysine-specific demethylase-1 (LSD1) and mammalian target of rapamycin (mTOR) via RN-1 and sirolimus, respectively, reduced mitochondrial retention with a concomitant reduction in ROS in RBCs from SCD mice [110]. The inhibition of mTORC also led to the induction of autophagy due to an increase in *ULK1* expression, a decrease in the α-globin chain, and an increase in HbF in β-thalassemia patients [111].

Mitoxantrone, an FDA-approved antitumor agent, has been shown to improve ineffective erythropoiesis in a β-thalassemia mouse model, possibly through mitochondrial pathways. This effect may be partly mediated by restoring mitochondrial homeostasis in reticulocytes via mitophagy induction, though further research is needed to explore this [112]. Impaired mitochondrial clearance is a significant contributor to the pathophysiology of thalassemia, leading to oxidative stress, anemia, and ineffective erythropoiesis. Strategies that enhance mitophagy, reduce oxidative damage, and restore mitochondrial function show promise for improving outcomes in thalassemia patients.

Moreover, mitochondria-targeted antioxidants like MitoQ are designed to specifically target and neutralize reactive oxygen species (ROS) within mitochondria. By focusing their antioxidant effects in mitochondria, these compounds aim to reduce oxidative damage associated with various diseases and aging. MitoQ treatment has been found to lower mtROS levels, alleviating oxidative stress and improving mitochondrial function in thalassemia models. Additionally, MitoQ has been shown to restore the quiescence and functionality of hematopoietic stem cells (HSCs) in thalassemia models, supporting a balance between glycolysis and oxidative phosphorylation, which is vital for proper HSC function [113]. Ongoing exploration of the molecular mechanisms behind mitochondrial dysfunction and clearance will be essential for developing targeted therapeutic interventions.

Recent studies have highlighted PGC-1α as a potential target for stimulating fetal hemoglobin (HbF) production, offering promising therapeutic prospects for hemoglobinopathies. The pharmacological activation of PGC-1α using small molecules like SR-18292 and ZLN005 has been shown to boost HbF expression in human erythroid cells and improve disease outcomes in transgenic mouse models [23]. In SCD mouse models treated with these compounds, there was a significant reduction in sickled red blood cells and a decrease in disease-related complications [114]. Additional research and clinical trials are needed to fully determine the effectiveness and safety of PGC-1α activators in human patients.

**Figure 3:**
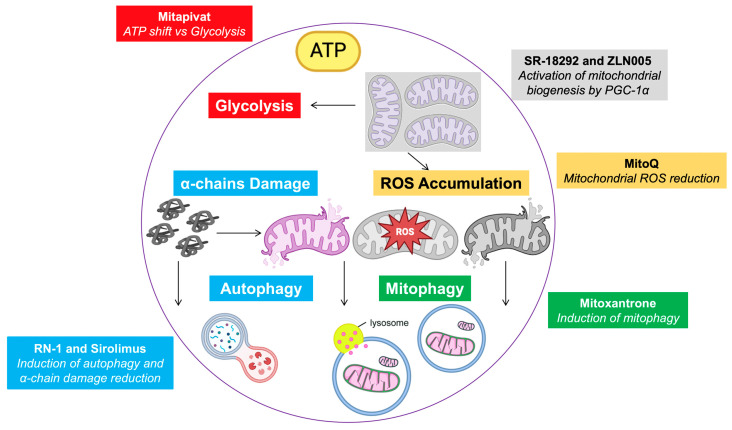
Overview of treatments and mitochondrial targets.

An adequate understanding of the processes regulating normal and disordered erythropoiesis has important implications for therapeutic interventions. In this context, the evaluation of metabolic pathways involving mitochondria as potential targets for treating β-thalassemia or other hematologic disorders associated with ineffective erythropoiesis is of interest (Figure 3).

## 8. Conclusions

To summarize, while thalassemia is primarily a disorder of hemoglobin production, mitochondrial dysfunction due to oxidative stress, iron overload, and energy demands plays a significant role in the pathology of the disease, contributing to its complications. The end stages of the erythroid cell maturation process include nuclear expulsion followed by the clearance of mitochondria and other organelles to generate mature RBCs. If these processes are improperly regulated, apoptosis may occur, resulting in ineffective erythropoiesis. Studies have suggested that mitochondria actively participate in erythroid cell maturation, erythroblast enucleation, and RBC production. Thus, mitochondria may serve as a potential target for developing new drugs to treat β-thalassemia.
